# Targeting Glutathione and Cystathionine β-Synthase in Ovarian Cancer Treatment by Selenium–Chrysin Polyurea Dendrimer Nanoformulation

**DOI:** 10.3390/nu11102523

**Published:** 2019-10-19

**Authors:** Inês Santos, Cristiano Ramos, Cindy Mendes, Catarina O. Sequeira, Catarina S. Tomé, Dalila G.H. Fernandes, Pedro Mota, Rita F. Pires, Donato Urso, Ana Hipólito, Alexandra M.M. Antunes, João B. Vicente, Sofia A. Pereira, Vasco D. B. Bonifácio, Sofia C. Nunes, Jacinta Serpa

**Affiliations:** 1 CEDOC, Chronic Diseases Research Centre, NOVA Medical School|Faculdade de Ciências Médicas, Universidade NOVA de Lisboa, Campo dos Mártires da Pátria, 130, 1169-056 Lisboa, Portugal; ips.ines95@gmail.com (I.S.); ramos.cristiano.93@gmail.com (C.R.); cindymendes8@gmail.com (C.M.); catarina.sequeira@nms.unl.pt (C.O.S.); ursodonato@gmail.com (D.U.); anaritac.hipolito@gmail.com (A.H.); sofia.pereira@nms.unl.pt (S.A.P.); asofianunes5@gmail.com (S.C.N.); 2 Instituto Português de Oncologia de Lisboa Francisco Gentil (IPOLFG), Rua Prof Lima Basto, 1099-023 Lisboa, Portugal; 3 Instituto de Tecnologia Química e Biológica António Xavier (ITQB NOVA), Avenida da República (EAN), 2780-157 Oeiras, Portugal; ctome@itqb.unl.pt (C.S.T.); dalilagfhf@itqb.unl.pt (D.G.H.F.); jvicente@itqb.unl.pt (J.B.V.); 4 CQFM-IN and Institute for Bioengineering and Biosciences, Instituto Superior Técnico, Universidade de Lisboa, Avenida Rovisco Pais, 1049-001 Lisboa, Portugal; pedromb.mota@gmail.com (P.M.); ritafpires@tecnico.ulisboa.pt (R.F.P.); vasco.bonifacio@tecnico.ulisboa.pt (V.D.B.B.); 5 Centro de Química Estrutural, Instituto Superior Técnico, ULisboa, Avenida Rovisco Pais, 1049-001 Lisboa, Portugal; alexandra.antunes@tecnico.ulisboa.pt

**Keywords:** ovarian cancer, platinum drugs, glutathione (GSH), cysteine, cystine/glutamate antiporter system Xc- (xCT), cystathionine β-synthase (CBS), hydrogen sulfide (H_2_S), selenium chrysin (SeChry), folate-targeted polyurea dendrimers

## Abstract

Ovarian cancer is the main cause of death from gynecological cancer, with its poor prognosis mainly related to late diagnosis and chemoresistance (acquired or intrinsic) to conventional alkylating and reactive oxygen species (ROS)-generating drugs. We and others reported that the availability of cysteine and glutathione (GSH) impacts the mechanisms of resistance to carboplatin in ovarian cancer. Different players in cysteine metabolism can be crucial in chemoresistance, such as the cystine/glutamate antiporter system Xc (xCT) and the H_2_S-synthesizing enzyme cystathionine β-synthase (CBS) in the pathway of cysteine catabolism. We hypothesized that, by disrupting cysteine metabolic flux, chemoresistance would be reverted. Since the xCT transporter is also able to take up selenium, we used selenium-containing chrysin (SeChry) as a plausible competitive inhibitor of xCT. For that, we tested the effects of SeChry on three different ovarian cancer cell lines (ES2, OVCAR3, and OVCAR8) and in two non-malignant cell lines (HaCaT and HK2). Results showed that, in addition to being highly cytotoxic, SeChry does not affect the uptake of cysteine, although it increases GSH depletion, indicating that SeChry might induce oxidative stress. However, enzymatic assays revealed an inhibitory effect of SeChry toward CBS, thus preventing production of the antioxidant H_2_S. Notably, our data showed that SeChry and folate-targeted polyurea dendrimer generation four (SeChry@PURE_G4_-FA) nanoparticles increased the specificity for SeChry delivery to ovarian cancer cells, reducing significantly the toxicity against non-malignant cells. Collectively, our data support SeChry@PURE_G4_-FA nanoparticles as a targeted strategy to improve ovarian cancer treatment, where GSH depletion and CBS inhibition underlie SeChry cytotoxicity.

## 1. Introduction

Ovarian cancer is the leading cause of death from gynecologic cancer [1,2,3]. This high mortality is mainly due to late diagnosis and chemotherapy resistance [4,5,6]. Lack of selective anti-cancer therapy prompts the use of comprehensive oxidative/alkylating drugs. Ovarian cancer is not an exception, and carboplatin is applied as a neoadjuvant, adjuvant, and palliative therapy, alone or combined with taxanes [1,7,8]. The mechanisms of action of platinum drugs are based on the establishment of adducts of DNA and proteins along with the generation of intracellular reactive oxygen species (ROS), leading to cell injury and death [9,10]. Chemoresistance against oxidative/alkylating drugs is associated with alterations in glutathione (GSH) and cysteine dynamics, as well as in cystathionine β-synthase (CBS)-catalyzed hydrogen sulfide (H_2_S) biosynthesis [9,11,12]. GSH is the most abundant low-molecular-weight thiol synthesized in cells, playing a crucial role in the drug detoxification process, consequently allowing cells to evade cell death [13,14,15,16,17,18,19]. Cysteine is the rate-limiting substrate in GSH production [20,21]^,^ and it is also linked to ovarian cancer progression by metabolomics and in vitro studies [22,23]. Our team showed that high GSH levels underlie ovarian cancer chemoresistance to carboplatin and that re-sensitization was accomplished by inhibiting GSH synthesis with buthionine sulfoximine (BSO) [24]. Furthermore, our previous results showed that the clear cell ovarian cancer histotype relies on cysteine metabolism to adapt to stressful metabolic conditions, such as hypoxia [25]. Additionally, we previously reported cysteine concentration (total and protein bound) in peripheral blood as a promising marker for screening, early diagnosis, outcome, and follow-up of ovarian cancer [25]. The overexpression of CBS associated with H_2_S generation was reported in several ovarian cancer cell lines and tumor models and was proposed to contribute to mitochondrial morphogenesis reprogramming [26], energy metabolism regulation [27], and deregulation of lipid metabolism [26], thereby promoting tumor growth and chemoresistance [12]. Indeed, CBS inhibition may afford anti-tumor activity and abrogation of chemoresistance via attenuated GSH synthesis and nuclear metallothionein expression [12,28].

Considering that cysteine is a substrate of CBS and a precursor of GSH, its import must be a crucial step in H_2_S generation and GSH synthesis. The cystine/glutamate antiporter system Xc- (xCT) is pivotal in the exchange of cystine (dimeric cysteine oxidized form) and glutamate, contributing to cellular homeostasis [23,29,30]. xCT is codified by the soluble carrier protein 7A11-encoding gene (*SLC7A11*). Both in physiological conditions and in cancer, xCT expression increases upon oxidative stress, enhancing cyst(e)ine influx, which in turn increases GSH synthesis and accounts for chemoresistance [31,32,33,34]. Therefore, efforts to develop compounds capable of interfering with xCT expression or function are a major point of interest [34,35]. Sulfasalazine and erastin were tested with this purpose, [31,36,37,38] although their action is not xCT-specific [36,39]. Herein, we hypothesized that a selenium analogue could be a valuable alternative to directly deplete GSH by inhibiting xCT.

Selenium (Se) is an essential element required for the maintenance of cellular redox balance [40,41] by being a cofactor of mammalian enzymes such as glutathione peroxidase [40,42]. In addition, selenium compounds are taken up by cyst(e)ine transporters [40,43,44], and some studies demonstrated that selenium itself exerts anti-tumoral activity [45,46]. In accordance, in small-cell lung cancer cell lines, xCT activity was related to higher selenium salt (selenite) sensitivity [47], revealing the possible use of xCT as a mediator of therapies based on selenium.

Altogether, these observations raised the hypothesis that selenium compounds are good candidates to impose a competitive inhibition of cyst(e)ine transport into the cell. In this work, we used selenium–chrysin (SeChry), a compound reported to display anti-tumoral and anti-oxidant properties [48,49,50]. SeChry was tested in two different ovarian cancer cell lines (ES2 and OVCAR3), and in non-malignant keratinocytes (HaCaT) and proximal tubule epithelial cells (HK2), in order to unravel its impact on cysteine uptake and carboplatin resistance, as well as its specificity against malignant cells.

## 2. Materials and Methods

### 2.1. Cell Culture

Three human ovarian cancer cells lines were used, serous carcinoma (OSC) cell lines (OVCAR3 HTB-161™ and OVCAR8 CVCL_1629^TM^) and a clear cell carcinoma (OCCC) cell line (ES2 CRL-1978™). Additionally, an immortal keratinocyte cell line (HaCaT PCS-200-011™) and a proximal tubular line derived from normal kidney (HK2 CRL- 2190™) were also tested. All the cell lines were obtained from American Type Culture Collection (ATCC) (Manassas, VA, USA). ES2, OVCAR3, OVCAR8, and HaCaT cell lines were cultured in Dulbecco’s modified Eagle medium (DMEM; 41965-039, Gibco, Life Technologies, Waltham, MA, USA) and HK2 in Dulbecco’s modified Eagle medium/Nutrient Mixture F-12 (DMEM-F12; 11320-033, Gibco, Life Technologies). All culture media were supplemented with 10% fetal bovine serum (FBS; S 0615, Merck, Burlington, VT, USA), 1% Antibiotic-Antimycotic (AA; P06-07300, PAN Biotech, Aidenbach, Germany) and 50 µg/mL gentamicin (15750-060, Gibco, Life Technologies). Cells were maintained in a humidified environment of 5% CO_2_ at 37 °C, until reaching approximately 75–100% optical confluence. Cells were detached with 0.05% trypsin–ethylenediaminetetraacetic acid (EDTA; 25300-054, Invitrogen, Thermo Fisher Scientific, Waltham, MA, USA) at room temperature (RT) for approximately 5 min. In cell death assays, cells in suspension in the culture media (supernatant) were analyzed together with adherent cells after trypsin harvesting. In all the other experiments, only viable adherent cells were analyzed.

### 2.2. Quantitative Real-Time PCR

ES2 and OVCAR3 cells (2 × 10^5^ cells/mL) were seeded in 12-well plates (1 mL/well) and cultured in control condition and exposed to cysteine (0.402 mM; 102839, Merck), carboplatin (0.025 mg/mL; IPO’s Pharmacy, Lisbon, Portugal), and cysteine combined with carboplatin. Prior to the addition of the experimental conditions, cells were synchronized under starvation (culture medium without FBS) for 8 h. 

The cells were collected after 16 h of experimental conditions, and RNA extraction was executed using RNeasy Mini Extraction kit (74,104, Qiagen, Hilden, Germany). The complementary DNA (cDNA) synthesis from 1 µg of RNA was performed using SuperScript II Reverse Transcriptase (18080e44, Invitrogen, Waltham, MA, USA). Quantitative real-time PCR was performed using SYBR Green PCR Master Mix (4309155, Applied Biosystems, Waltham, MA, USA), according to the manufacturer’s protocol. Real-time PCR was carried out in a Lightcycler® 480 System instrument (05015243001, Roche, Basel, Switzerland). The *xCT/SLC7A11* expression was quantified (forward 5’–GGTCCTGTCACTATTTGGAGC–3’ and reverse 5’–GAGGAGTTCCACCCAGACTC–3’), and hypoxanthine–guanine phosphoribosyltransferase 1 (*HPRT1*) was used as a housekeeping gene (forward 5’–TGACACTGGCAAAACAATG–3’ and reverse 5’–GGTCGTTTTTCACCAGCAA–3’).

### 2.3. Western Blotting

ES2 and OVCAR3 cells (2.5 × 10^5^ cells/mL) were seeded in six-well plates (2 mL/well), cultured in control conditions, and exposed to l-cysteine (0.402 mM; 102839, Merck), carboplatin (0.025 mg/mL; IPO’s Pharmacy), and cysteine combined with carboplatin for 16 h. Prior to the addition of the experimental conditions, cells were synchronized under starvation.

For total protein extraction, cell pellets were lysed using radio-immunoprecipitation assay (RIPA) buffer. Protein concentration was determined spectrophotometrically (595 nm) via the Bradford method, using the Bio-Rad protein assay reagent (500-0006, Bio-Rad, Hercules, RI, USA). After the addition of loading buffer containing 10% SDS, 0.5% bromophenol blue in Tris-HCl (pH 6.8), and 10% β-mercaptoethanol (M3148, Sigma, St. Louis, MO, USA), samples (range of 50–100 µg) were boiled at 95–100 °C for 10 min. Samples were analyzed in 12% PAGE (polyacrylamide gel electrophoresis). Proteins were transferred to an Immuno-Blot® polyvinylidene fluoride (PVDF) membrane with a Trans-Blot® Turbo^TM^ Blotting system, posteriorly incubated with rabbit anti-human xCT (ab175186-Abcam, 1:1000) overnight at 4 °C, and further incubated with secondary antibody immunoglobulin G (IgG)-conjugated horseradish peroxidase (HRP; anti-rabbit, 1:5000, 31460, Thermo Scientific Scientific), for 2 h at RT. After these incubations, specific bands were detected using enhanced chemiluminescence (ECL) Western blotting substrate (SuperSignal® West Pico Chemiluminescent Substrate, 34080, Thermo Scientific) in a ChemiDoc XRS System (Bio-Rad) with Image Lab software. The detection of the endogenous control (β-actin) was also performed (mouse anti-human β-actin; 1:5000, A5441, Sigma and secondary HRP-conjugated; anti-mouse, 1:5000, 31430, Thermo Scientific). Bands were quantified using Image J software (rsb.info.nih.gov/ij/).

### 2.4. Immunofluorescence

To analyze the effects of cysteine and/or carboplatin on nuclear factor erythroid 2-related factor 2 (Nrf2) expression and SeChry effect on xCT expression, lamellas were inserted in wells, coated with 0.2% gelatin (G-1890, Sigma Aldrich, St. Louis, MO, USA); then, cells (2 × 10^5^ cells/mL), seeded in 24-well plates (500 µL/well), were submitted to control, cysteine (0.402 mM; 102839, Merck), carboplatin (0.025 mg/mL; IPO’s Pharmacy), and cysteine combined with carboplatin for 16 h. In SeChry assays, cells were exposed to SeChry (19 µM, a concentration above the half maximal effective concentration (EC_50_; see Figure S1) for 24 h and then, without removing SeChry, exposed to the other experimental conditions (cysteine and carboplatin) for another 24 h. After this, cells were fixed with 4% paraformaldehyde (104003, Merck Millipore, Burlington, VT, USA) for 15 min at 4 °C. Posteriorly, in order to permeabilize cells, 0.1% saponin in phosphate-buffered saline PBS (1×) with 0.5% bovine serum albumin (BSA) was applied to the cells for 15 min at RT. Cells were then incubated with primary antibody (Rabbit anti-human xCT, ab175186-Abcam; Rabbit anti-human Nrf2, ab62352-Abcam; 1:100 diluted in 0.1% saponin in PBS (1×) with 0.5% BSA), 3 h at RT, followed by incubation with the secondary antibody Alexa Fluor® 488 anti-rabbit (A-11034, Invitrogen) for 1 h at RT. Slides were mounted in VECTASHIELD media containing DAPI (4′-6-diamidino-2-phenylindole) (H-1200, Vector Labs, Burlingame, CA, USA). The analysis was performed by standard fluorescence microscopy using a Zeiss Imajer.Z1 AX10 microscope. Images were acquired and processed with CytoVision software.

### 2.5. Cell Death Analysis by Flow Cytometry

To examine cell death resultant from carboplatin (0.025 mg/mL; IPO’s Pharmacy) exposure with and without cysteine (0.402 mM; 102839, Merck) or the carboplatin cysteinyl-*S*-conjugate (the metabolite generated upon the catabolism of GSH–carboplatin adduct) [51], cells (2 × 10^5^ cells/mL) were seeded in 24-well (500 µL/well) plates incubated for 16 h. In the case of the SeChry (19 µM, a concentration above EC_50_; see Figure S1) effect on cell death, an incubation of 48 h with SeChry was performed, and, in the last 24 h, cysteine and/or carboplatin were added. After experimental conditions, the supernatants were collected, and adherent cells were harvested with 0.05% trypsin–EDTA. Cells in the supernatant and trypsinized cells were joined and centrifuged at 255× *g* for 2 min. Cells were stained with 0.5 μL annexin V–fluorescein isothiocyanate (FITC) (640906, BioLegend, San Diego, CA, USA), in annexin V binding buffer 1×, and incubated at RT, in dark for 15 min. Samples were resuspended in 200 μL PBS (1×) with0.1% BSA and centrifuged at 255× *g* for 2 min. Cells were resuspended in 200 μL of annexin V binding buffer 1×, and 2.5 μL of propidium iodide (PI, 50 μg/mL; P4170, Sigma-Aldrich) was added 5 min prior to analysis. Afterward, samples were analyzed by flow cytometry (FACScalibur, Becton Dickinson). Data were analyzed using FlowJo 8.7 software (https://www.flowjo.com).

### 2.6. High-Performance Liquid Chromatography (HPLC)

The effect of SeChry on cysteine uptake and GSH content was tested in ES2 and OVCAR3 cells by HPLC with fluorescence detection (FLD). Both the extracellular and the intracellular thiols were assessed, as the total levels and total free levels. The levels of cysteine (Cys), glutathione (GSH), and cysteinyl-glycine (CysGly) were assessed according to Grilo and co-authors [52] adapted to cell culture. The detector was set at excitation and emission wavelengths of 385 and 515 nm, respectively. The mobile phase consisted of 100 mM acetate buffer (pH 4.5) and methanol (98:2 (*v*/*v*)). The analytes were separated in an isocratic elution mode for 20 min, at a flow rate of 0.6 mL/min. 

Cells (2.5 × 10^5^ cells/mL) were cultured in six-well plates (2 mL/well) for 24 h, without starvation and exposed to SeChry (19 µM, a concentration above EC_50_; see Figure S1). After this period, cells were incubated with cysteine (0.402 mM; 102839, Merck) for 30 min and 2 h, and were harvested with 0.05% trypsin–EDTA, centrifuged at 255× *g* for 2 min, rinsed twice in PBS (1×), and lysed with 120 µL PBS (1×) with 0.01% (*v*/*v*) Triton X-100. Cell lysates and supernatants were centrifuged at 10,600× *g* for 2 min. The supernatants and the lysates were stored at −80 °C.

### 2.7. Synthesis of SeChry

Selenium-containing chrysin (SeChry) was synthesized following a reported protocol [48]. After purification, the formation of the product was confirmed by ^1^H NMR. ^1^H NMR (CDCl_3_, 400 MHz) δ (ppm): 7.96 (2H, d, *J* = 8.0 Hz), 7.76 (1H, s), 7.61 (1H, t, *J* = 8.0 Hz), 7.52 (2H, t, *J* = 8.0 Hz), 6.51 (1H, d, *J* = 4.0 Hz), 6.46 (1H, d, *J* = 4.0 Hz).

SeChry is stable for several months if stored at 4 °C under inert atmosphere. Partial deselenization may occur for storage at room temperature in the presence of oxygen (up to 30% in a two-month period). No degradation was observed in the culture medium under the experimental conditions of the performed assays (purity checked by CHCl_3_ extraction from the medium followed by NMR analysis).

Since SeChry is not water-soluble, fresh SeChry solutions were prepared for all the assays. For each experiment, a stock solution of 1 M was prepared in 100% dimethyl sulfoxide (DMSO). Afterward, the appropriate intermediate solutions were also prepared in 100% DMSO in order to use the final desired concentrations of SeChry with a final concentration of 0.2% DMSO in the cell culture medium. Accordingly, 0.2% was used in the DMSO control condition.

### 2.8. Synthesis of Folate-Targeted Polyurea Dendrimer Generation Four (PURE_G4_-FA) Nanoparticles

Folate-targeted polyurea dendrimer generation four (PURE_G4_-FA) was prepared by reacting polyurea dendrimer generation four (PURE_G4_), obtained using our supercritical-assisted polymerization protocol [53], with activated folic acid succinic ester (FA-NHS). FA-NHS was synthesized following the literature [54]. Typically, in a round-bottom flask, 250 mg (0.566 mmol) of folic acid (FA) was dissolved in DMSO (2.75 mL). After the addition of 130.8 mg (1.137 mmol) of *N*-hydroxysuccinimide (NHS), 128.5 mg (0.623 mmol) of *N,N′*-dicyclocarbodiimide (DCC), and 0.15 mL (1.082 mmol) of triethylamine (TEA), the reaction was stirred at RT overnight in the dark. The product was precipitated and washed several times with diethyl ether. After drying under vacuum, FA-NHS was obtained as a yellow powder (263.4 mg) in 86.4% yield. ^1^H NMR (400 MHz, DMSO-*d6*) δ (ppm): 8.64 (1H, s), 7.63 (2H, d, *J* = 8.0 Hz), 6.64 (2H, d, *J* = 8.0 Hz), 4.49 (2H, s), 4.28 (1H, s), 2.54 (4H, s), 2.29 (1H, s), 2.03 (1H, s), 1.93 (1H, s).

Next, FA-NHS was conjugated with PURE_G4_ (via NH_2_ surface groups) to obtain PURE_G4_-FA. In a 25-mL round-bottom flask, 100 mg (0.0127 mmol) of PURE_G4_ was dissolved in 5.0 mL of DMSO. To this solution, 13.7 mg (0.0254 mmol) of FA-NHS and 6.9 μL (0.0510 mmol) of TEA were added. The reaction was stirred at RT overnight in the dark. Next, TEA excess was removed on the rotary evaporator, and diethyl ether was added. The obtained precipitate was dried under vacuum, and PURE_G4_-FA was obtained as yellow oil in 93.9% yield. Via NMR, it was found that two molecules of folic acid were conjugated to the surface of PURE_G4_. ^1^H NMR (400 MHz, D_2_O) δ (ppm): 8.64 (2H, s), 7.70 (4H, bs), 6.86 (4H, d, *J* = 8.0 Hz), 4.61 (2H, s), 3.54–3.00 (180H, m), 2.96–2.40 (462H, m).

### 2.9. Preparation of SeChry@PURE_G4_-FA Nanoparticles

SeChry was encapsulated in PURE_G4_-FA nanoparticles following a modified protocol [55]. Briefly, a CHCl_3_ solution (0.5 mL) of SeChry (6.5 mg) was added to an aqueous solution (2 mL) of PURE_G4_-FA (125 mg). Next, CHCl_3_ was removed in a rotary evaporator and the mixture allowed stirring at RT overnight. Then, the aqueous solution was extracted with CHCl_3_ to remove non-encapsulated or surface-bound SeChry. No SeChry was found in the CHCl_3_ extracts (control by thin-layer chromatography (TLC)), thus confirming a full encapsulation. The release profile followed the usual profile reported for this nanodelivery system [55].

### 2.10. Inhibition of H_2_S-Synthesizing Enzymes by SeChry

The ability of SeChry to inhibit the human H_2_S-synthesizing enzymes was evaluated by activity assays carried out with isolated recombinant cystathionine β-synthase (tCBS, a catalytically competent truncated version lacking the s-adenosyl-l-methionine-responsive regulatory C-terminal domain), cystathionine γ-lyase (CSE), and 3-mercaptopyruvate sulfurtransferase (MST). Proteins were produced and isolated as reported [56]. The H_2_S-synthesizing assays were performed, employing both the fluorometric probe 7-azido-4-methylcoumarin (AzMc) and the colorimetric methylene blue (MB) method [56]. Fluorescence and absorbance readings were recorded in a FLUOstar Optima (BMG Labtech) plate reader. Whereas the AzMc method was performed in the micro-plate format as described [56], the MB method was carried out in Eppendorf tubes to allow centrifugation to remove precipitated protein. SeChry solutions were prepared and diluted in 100% DMSO and added to all reactions in a 1/100 dilution. Reactions and the respective controls containing all reagents were performed in triplicate.

### 2.11. Statistical Analysis

Statistical analyses were performed in GraphPad Prism 7.0 software (www.graphpad.com). Data are presented as means ± SD. Assays were performed with at least biological three replicates. For comparisons of two groups, a two-tailed unpaired *t*-test was used. For more than two groups, one-way and two-way analyses of variance (ANOVA) with Tukey’s multiple comparisons post hoc test were used. Statistical significance was established as *p* < 0.05.

## 3. Results

### 3.1. Cysteine Partially Abrogates Platinum-Induced Cell Death and Modulates xCT Expression

**Figure 1:**
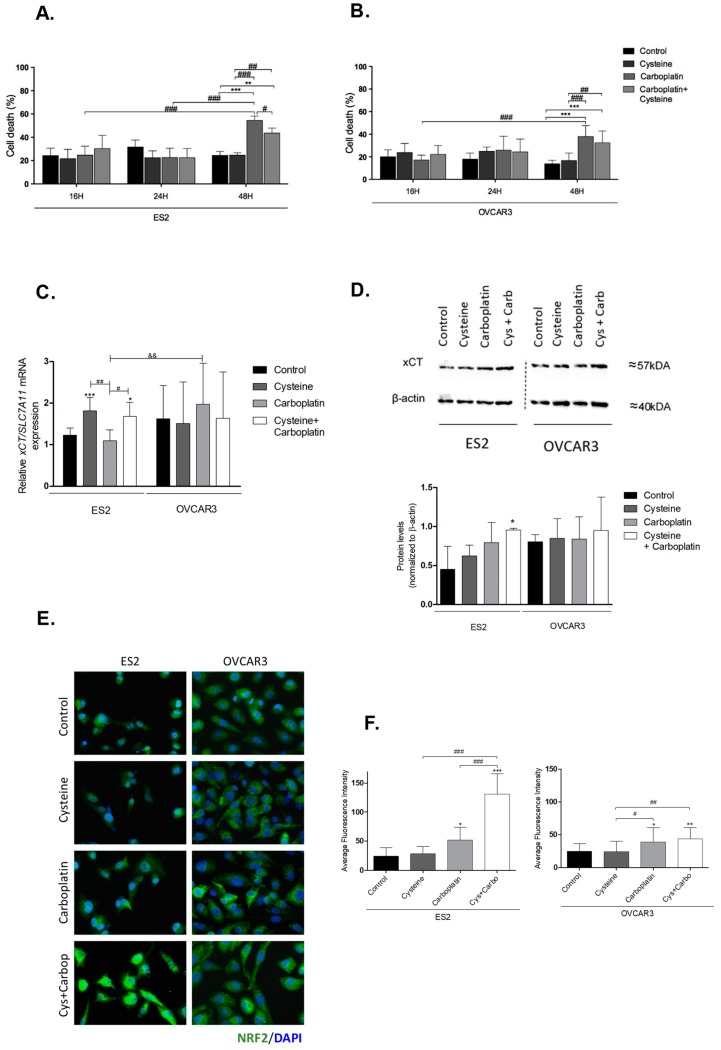
Putative involvement of cystine/glutamate antiporter system Xc (xCT) and nuclear factor erythroid 2-related factor 2 (Nrf2) in the protective effect of cysteine upon platinum salt exposure. ES2 and OVCAR3 cell lines were incubated with cysteine, carboplatin, and a combination of carboplatin with cysteine for 16 h (**A,B**). Cell death was determined by flow cytometry using annexin V fluorescein isothiocyanate (FITC) and propidium iodide (PI) staining. The *xCT*/soluble carrier protein 7A11-encoding gene (*SLC27A11*) messenger RA (mRNA) expression was analyzed by RT-qPCR (**C**). Hypoxanthine–guanine phosphoribosyltransferase (HPRT) was used as the housekeeping gene. The xCT protein levels were assessed by Western blotting; β-actin was used as the house-keeping protein (**D**). Nrf2 protein levels were measured by immunofluorescence (**E,F**). Results are shown as means ± SD; * *p* ≤ 0.05, ** *p* ≤ 0.01, *** *p* ≤ 0.001 (**A**–**D**, and **F**), and represents statistical significance in relation to control conditions, # *p* ≤ 0.05, ## *p* ≤ 0.01, ### *p* ≤ 0.001 represents statistical significance between conditions, and && *p* ≤ 0.01 represents statistical significance between conditions among cell lines (**A**–**D**, and **F**).

Considering our previous observation that cysteine contributes to ovarian cancer cell resistance to platinum drugs [25,57], we aimed to further evaluate the cysteine protective contribution at different time points. At 48 h, cysteine was significantly protective to ES2 cells against carboplatin-induced cell death (Figure 1A). In OVCAR3 cells, at 48 h, there was also a tendency for a protective effect of cysteine upon carboplatin exposure, albeit not significant (Figure 1B). 

As xCT is important to cyst(e)ine uptake (considering that, extracellularly, cysteine is converted to the oxidized form cystine), we analyzed the expression of *xCT/SLC7A11* upon exposure to cysteine and carboplatin. Cysteine significantly upregulated *xCT/SLC7A11*, with and without carboplatin, in the ES2 cell line but not in OVCAR3 (Figure 1C). Considering protein levels, there was a trend to increased xCT levels upon carboplatin exposure in ES2. In cells exposed solely to carboplatin, the levels tended to be higher but without statistical significance, whereas, in cells exposed to carboplatin plus cysteine, xCT levels were significantly increased (Figure 1D). In OVCAR3 cells, no significant differences were observed in xCT protein levels (Figure 1D). 

Collectively, these results show that cysteine has a protective effect against carboplatin, mainly in ES2 cells with a longer period of carboplatin exposure. Moreover, both cysteine and carboplatin can modulate xCT expression in ES2 at the transcriptional and translational levels, respectively. 

### 3.2. Cysteine and Carboplatin Modulate the Expression of Nrf2

Nrf2 is a transcription factor involved in redox homeostasis, which is overexpressed upon oxidative stress, and it is described as the main activator of *xCT/SLC7A11* [29,58,59,60]. Immunofluorescence analysis showed that carboplatin exposure significantly upregulated Nrf2 levels in both cell lines (Figure 1E,F). In ES2, a synergy between the effects of cysteine and carboplatin was observed given their cumulative effect on Nrf2 levels. 

### 3.3. SeChry Induces Cell Death and xCT Expression in Ovarian Cancer Cells

**Figure 2:**
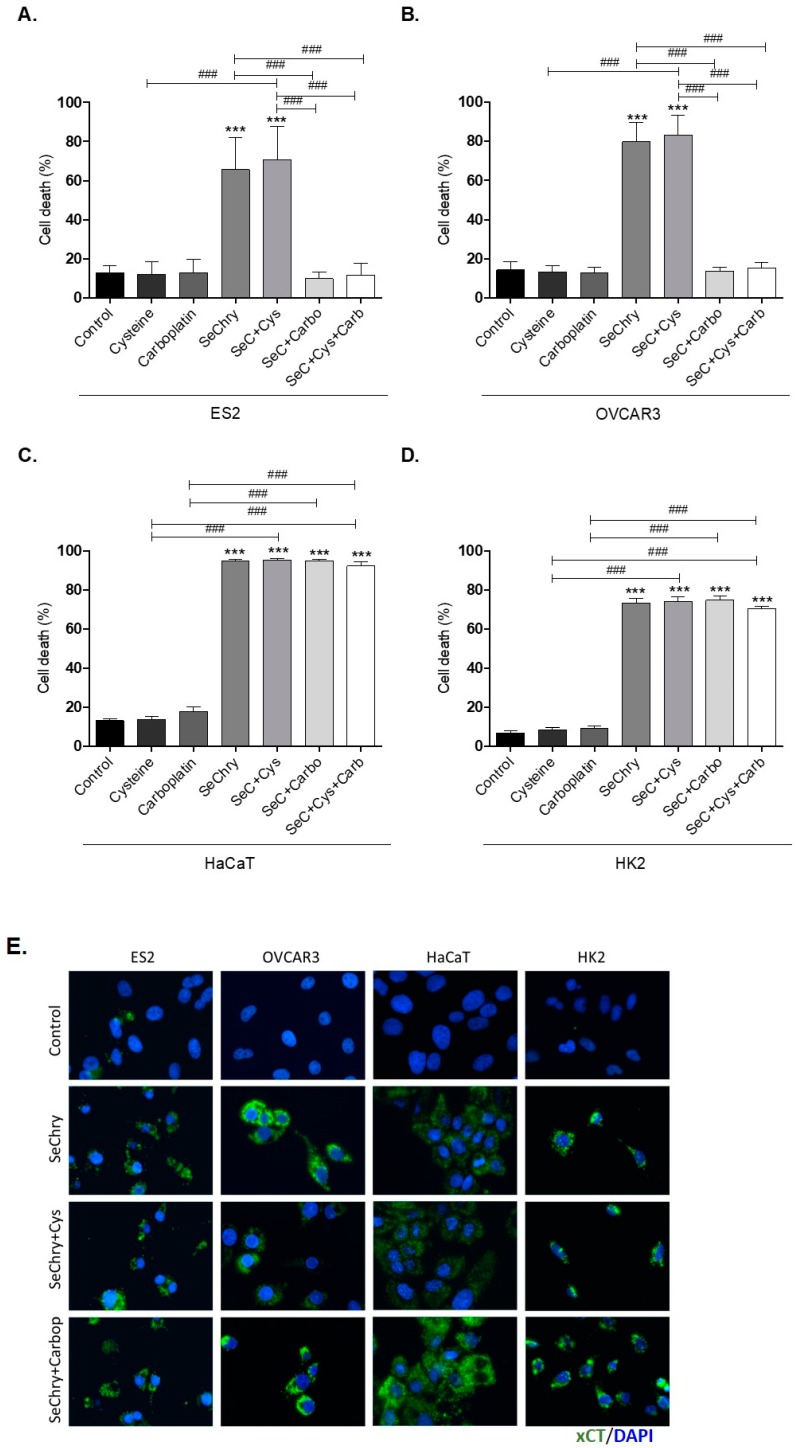
Selenium–chrysin (SeChry) induces cell death and xCT expression. All cell lines (ES2, OVCAR3, HaCaT, and HK2) were exposed to dimethyl sulfoxide (DMSO; control), cysteine, carboplatin, SeChry, and SeChry combined with cysteine and/or carboplatin for 24 h and 48 h of SeChry exposure (**A**–**D**). Cell death was analyzed by flow cytometry using annexin V–FITC and propidium iodide (PI) staining. The xCT protein levels were evaluated by immunofluorescence (**E**). Results are shown as means ± SD; *** *p* < 0.001, and represents statistical significance in relation to control conditions, and ### *p* ≤ 0.001 represents statistical significance between conditions (one-way ANOVA, Tukey post-test).

SeChry was previously reported as having a cytotoxic effect on cancer cells with the ability to overcome cisplatin resistance and multiple drug resistance (MDR) [48,49,50]. As a selenium-containing small molecule, we hypothesized that it could function as a competitor of cyst(e)ine for xCT binding and transport. Considering that the ability of cyst(e)ine uptake contributes to chemoresistance, blocking xCT with SeChry could revert this chemoresistance phenomenon. Thus, we firstly aimed to disclose the impact of SeChry on cell death upon cysteine and/or carboplatin exposure. For this, SeChry was tested (48 h) with and without cysteine or carboplatin exposure (24 h). The effect of carboplatin was tested for 24 h, since, at this time point, carboplatin does not significantly affect cell viability, which allowed us to analyze the effect of SeChry on cell death and possible synergistic or antagonistic effects. In addition to ES2 and OVCAR3, we also tested this compound on HaCaT, a keratinocyte cell line, due to its recognized sensibility to compounds containing selenium, and on HK2, a kidney tubular cell line, due to the susceptibility of renal cells to platinum salt injury. In ES2 and OVCAR3, SeChry alone or combined with cysteine showed increased cell death levels compared to control conditions (Figure 2A,B). However, SeChry toxicity was abrogated by carboplatin, independently of the presence of cysteine (Figure 2A,B). In turn, HaCaT and HK2 cell lines presented augmented cell death in all SeChry conditions (Figure 2C,D).

To test the effects of SeChry on *xCT/SLC7A11* expression, protein levels were assessed by immunofluorescence. Interestingly, in all cell lines, xCT expression increased upon exposure to SeChry alone or combined with cysteine or carboplatin (Figure 2E).

So far, results indicate that SeChry induces cell death and *xCT/SLC7A11* expression in both ovarian cancer cells and non-malignant cells. However, contrarily to what was expected, SeChry does not allow the chemoresistance reversion in ovarian cancer cells. Instead, SeChry cytotoxicity decreased upon carboplatin exposure.

### 3.4. SeChry Does Not Impair But Enhances Cysteine Uptake

**Figure 3:**
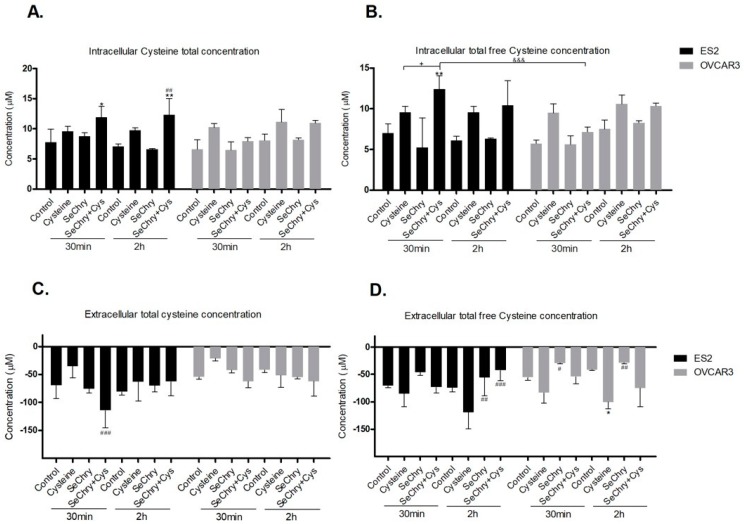
SeChry does not impair cysteine uptake. ES2 and OVCAR3 cell lines were exposed to DMSO (control), cysteine, SeChry, and SeChry combined with cysteine. After 24 h of SeChry exposure, cells were incubated with cysteine for 30 min and 2 h. High-performance liquid chromatography (HPLC) coupled to fluorescence detection (FLD) was used to measure (**A**) intracellular total cysteine concentration, (**B**) intracellular total free cysteine concentration, (**C**) total cysteine concentration in the culture media (supernatant), and (**D**) total free cysteine concentration in the supernatant. Results are shown as means ± SD; * *p* < 0.05, ** *p* < 0.01, and represents statistical significance in relation to control, # *p* < 0.05, ## *p* < 0.01, ### *p* < 0.001 represents statistical significance in relation to cysteine, “+” represents statistical significance between conditions at the same time point, and &&& *p* < 0.001 represents statistical significance between conditions among cell lines.

We hypothesized that SeChry induces cell death due to cysteine uptake impairment. To test this hypothesis, cells were exposed to SeChry with and without cysteine, and the intracellular and extracellular thiols levels of cell lysates and culture medium (supernatant) were determined by HPLC-FLD analysis. In both cell lines, SeChry did not impair cysteine uptake, as no significant differences were found both in intracellular (Figure 3A,B) and extracellular (Figure 3C,D) levels compared to the control. The only exception was the extracellular total free levels of cysteine in OVCAR3 cells, which suggested lower cysteine uptake; however, no significant differences in the intracellular total free levels of cysteine were observed in OVCAR3 cells (Figure 3B,D). Moreover, in ES2 cells and in the presence of cysteine, data indicate that SeChry enhances cysteine uptake, as increased total and free total intracellular cysteine levels (Figure 3A,B) and decreased total extracellular levels (Figure 3C) (*p* < 0.05) were observed, mainly upon 30 min of experimental conditions. In OVCAR3 and in the presence of cysteine, data suggest that SeChry does not enhance cysteine uptake, as no significant differences in intracellular (Figure 3A,B) and extracellular cysteine levels (Figure 3C,D) were observed among control and SeChry plus cysteine conditions. Overall, SeChry does not lead to cysteine uptake impairment; rather, it appears to enhance it, at least in ES2 cells.

### 3.5. SeChry Enhances Glutathione (GSH) Turnover

Intra- and extracellular GSH levels and extracellular levels of cysteinyl-glycine (Cys-Gly, a product of GSH degradation) were evaluated in the absence or presence of SeChry in order to evaluate the effect of SeChry on the dynamics of GSH synthesis and degradation.

**Figure 4:**
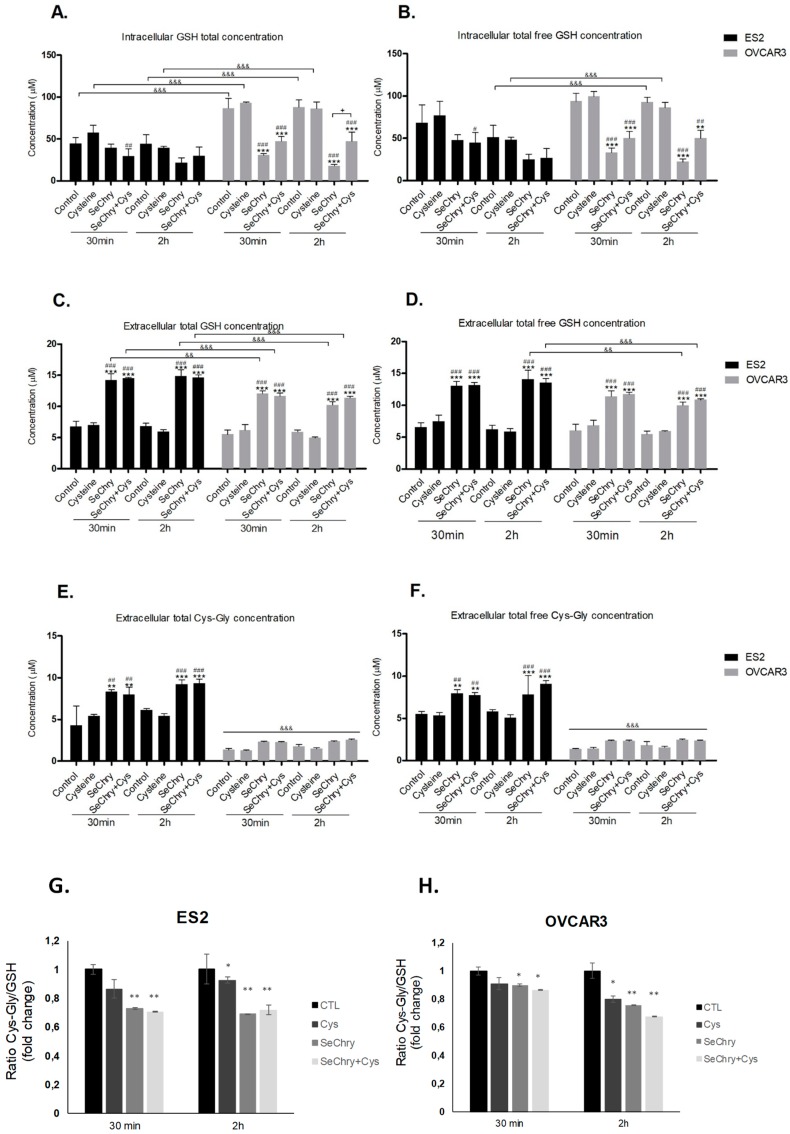
SeChry induces glutathione (GSH) turnover. ES2 and OVCAR3 cell lines were exposed to DMSO (control), cysteine, SeChry, and SeChry plus (+) cysteine; after 24 h of SeChry exposure, cells were incubated with cysteine for 30 min and 2 h. High-performance liquid chromatography (HPLC) coupled to fluorescence detection was used to measure (**A**) intracellular total GSH concentration, (**B**) intracellular total free GSH concentration, (**C**) total GSH concentration in the supernatant, (**D**) total free GSH concentration in the supernatant, (**E**) total cysteinyl-glycine (Cys-Gly) concentration in the supernatant, (**F**) total free GSH concentration in the supernatant, and the extracellular ratio of Cys-Gly/GSH in ES2 (**G**) and OVCAR3 (**H**) cells. Results are shown as means ± SD; * *p* < 0.05, ** *p* < 0.01, *** *p* < 0.001, and represents statistical significance in relation to control, “#” represents statistical significance in relation to cysteine, # *p* < 0.05, ## *p* < 0.01, ### *p* < 0.001represents statistical significance between conditions at the same time point, and && *p* < 0.01, &&& *p* < 0.001represents statistical significance between conditions among cell lines.

Compared to the control, SeChry led to decreased GSH content, mainly in OVCAR3 cells (Figure 4A,B). However, in the presence of cysteine, SeChry also decreased GSH content in ES2 cells (Figure 4A,B). Regarding the extracellular levels, SeChry increased the GSH levels (total and total free) independently of cysteine, in both cell lines (Figure 4C,D). Furthermore, the same dynamics were observed for Cys-Gly in ES2 but not in OVCAR3 cells (Figure 4E,F). The data support the fact that, in addition to not impairing cysteine uptake, SeChry prompts GSH degradation, which involves transport of GSH to the extracellular compartment where GSH is further degraded into the dipeptide Cys-Gly through γ-glutamyl-transpeptidase activity. The Cys-Gly/GSH ratio shows that SeChry stimulates GSH turnover in both ES2 and OVCAR3, with the same pattern (Figure 4G,H). 

### 3.6. SeChry Inhibits Cystathionine β-Synthase (CBS)

As SeChry did not impair or enhance cyst(e)ine uptake, we wondered whether its cytotoxic effects could be related to the H_2_S-synthesizing enzymes in the path of cysteine catabolism: cystathionine β-synthase (CBS), cystathionine γ-lyase (CSE), and 3-mercapto-pyruvate sulfurtransferase (MST) [61,62,63].

**Figure 5:**
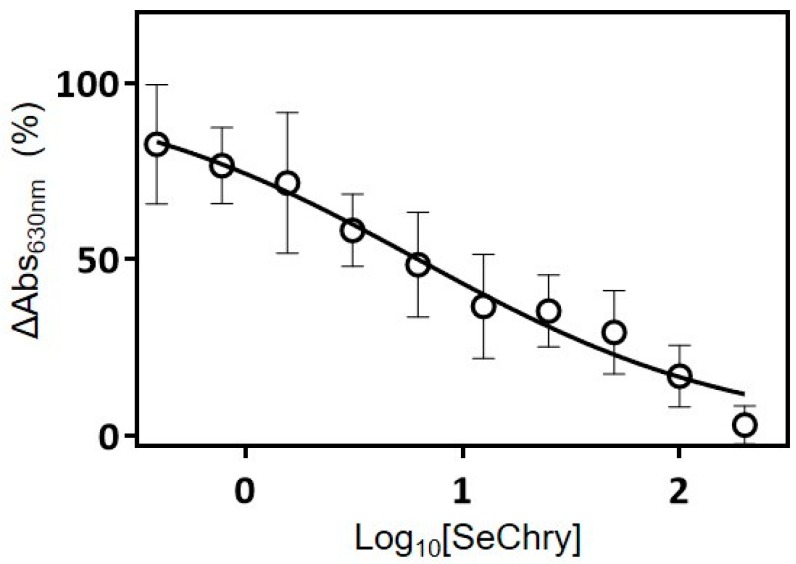
SeChry inhibits human cystathionine β-synthase H_2_S-synthesizing activity. Cystathionine β-synthase (CBS)-catalyzed H_2_S production detected by the methylene blue assay, measured as a function of SeChry concentration in the 0–200-µM range. Data from four independent experiments (triplicates of each reaction and respective control) were fitted to a log_10_[inhibitor] versus normalized response curve, yielding an apparent half maximal inhibitory concentration (IC_50_) of 6.3 ± 1.2 µM.

Initial attempts to employ the fluorometric AzMc probe to evaluate the inhibition of the human H_2_S-synthesizing enzymes by SeChry were hampered by the strong interference of SeChry with the fluorometric probe, which precluded measuring the enzymatic activity (data not shown). Therefore, we resorted to the colorimetric methylene blue method. By testing a single SeChry concentration of 200 µM, we observed that tCBS was completely inhibited by SeChry, whereas neither CSE nor MST were inhibited. The tCBS inhibition was then evaluated as a function of SeChry concentration in the 0–200-µM range (Figure 5), yielding an apparent half maximal inhibitory concentration (IC_50_) of 6.3 ± 1.2 µM.

### 3.7. SeChry Delivery by PURE_G4_-FA Nanoparticles Allows the More Specific Targeting of Ovarian Cancer Cells, While Reducing Cytotoxicity in Non-Malignant Cells

**Figure 6:**
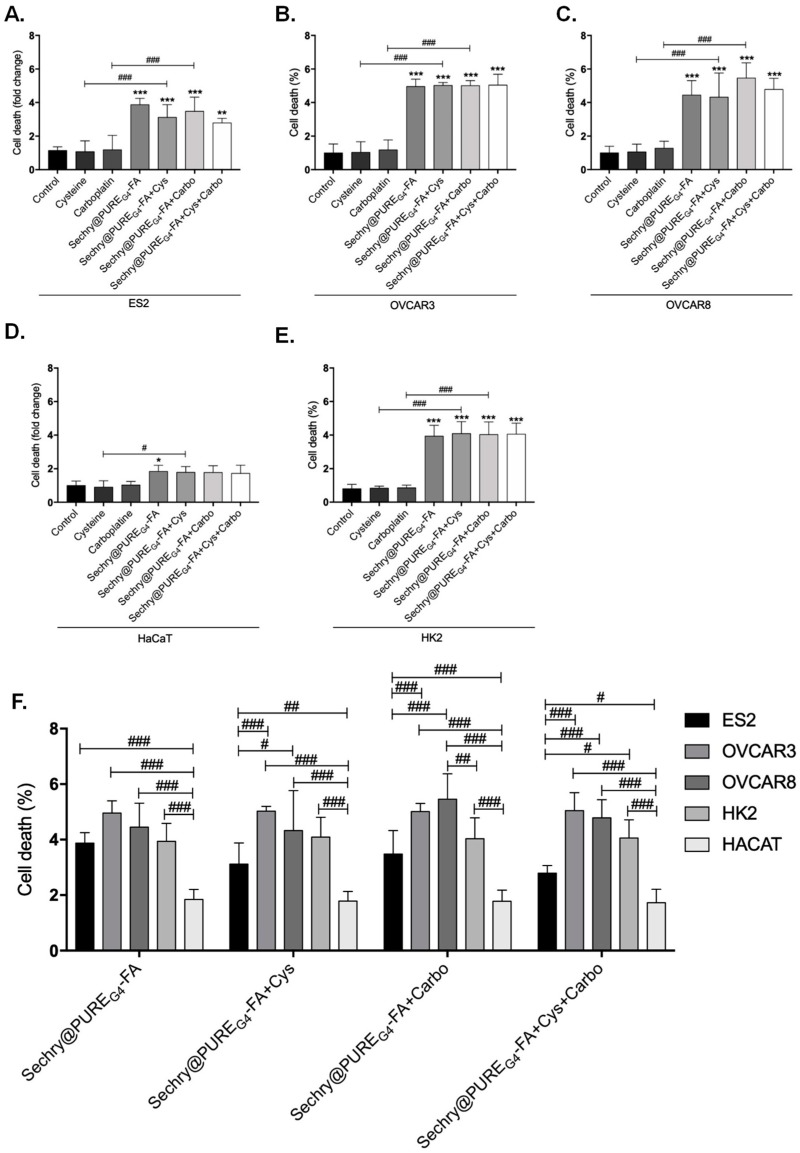
SeChry and folate-targeted polyurea dendrimer generation four (SeChry@PURE_G4_-FA) nanoparticles increase specificity to ovarian cancer cells. All cell lines (ES2, OVCAR3, OVCAR8, HaCaT, and HK2) were exposed to DMSO (control), cysteine, carboplatin, SeChry@PURE_G4_-FA, and SeChry@PURE_G4_-FA combined with cysteine and/or carboplatin for 24 h and 48 h of SeChry@PURE_G4_-FA exposure (**A**–**E**). Cell death was analyzed by flow cytometry using annexin V–FITC and propidium iodide (PI) staining. SeChry@PURE_G4_-FA and SeChry@PURE_G4_-FA combined with cysteine and/or carboplatin conditions were compared between cell lines (**F**). Results are shown as means ± SD; * *p* < 0.05, ** *p* < 0.01, *** *p* < 0.001, and represents statistical significance in relation to control conditions, and # *p* < 0.05, ## *p* < 0.01, ### *p* < 0.001represents statistical significance between conditions (one-way ANOVA, Tukey post-test).

Ovarian cancer cells express higher levels of folate receptor than normal cells [64], and folate receptor-targeted therapy is a way of directing nanoparticles to ovarian cancer cells both in vitro and in vivo [65]. Thus, as an attempt to improve the specificity of SeChry toward ovarian cancer cells, folate receptor-targeted delivery of SeChry was performed using SeChry@PURE_G4_-FA nanoparticles as a drug delivery system. Cells were exposed to encapsulated SeChry (SeChry@PURE_G4_-FA) alone or in combination with cysteine and/or carboplatin, and cell death was assessed by flow cytometry. Results showed that SeChry@PURE_G4_-FA maintained cytotoxicity for ovarian cancer cells (ES2, OVCAR3, and OVCAR8), though showing lower levels of cell death (Figure 6A–C). Notably, the previously observed reversion of SeChry toxicity upon carboplatin exposure in both ES2 and OVCAR3 cells (Figure 2A,B) was not observed with SeChry@PURE_G4_-FA nanoparticles (Figure 6A,B). In OVCAR8 cells, carboplatin also did not interfere with the cytotoxic effect of SeChry@PURE_G4_-FA nanoparticles (Figure 6C). Considering HaCaT and HK2 cells, strikingly, SeChry@PURE_G4_-FA nanoparticles presented lower toxicity (Figure 6D,E) as compared to free SeChry (Figure 2C,D). Importantly, we observed significantly higher levels of cell death in three ovarian cancer cell lines compared to non-malignant cells, mainly keratinocytes, upon SeChry@PURE_G4_-FA nanoparticle exposure (Figure 6F).

## 4. Discussion

In ovarian cancer, chemoresistance is a major hindrance for a cure. Thus, the identification of relevant players in the chemoresistance mechanisms is a key step to unveil the ovarian cancer response to therapeutics [8,14,66,67] In this study, in line with our previous observations [25,57], cysteine exhibited a protective role from carboplatin-induced cytotoxicity (Figure 1A,B). As oxidative/alkylating agents, platinum drugs are responsible for GSH decrease through direct chemical interactions, leading to the formation of GSH conjugates, as well as ROS [9,66,67]. Indeed, the combination of high GSH doses with platinum protects cells against cisplatin-induced toxicity [68]. Therefore, cysteine bioavailability is undoubtedly a motor for drug detoxification through both GSH synthesis and as a precursor of H_2_S generation.

The xCT expression is intimately correlated with chemoresistance, which relies on xCT overexpression and consequently increased cyst(e)ine uptake [23,69,70]. The role of xCT in cancer is extensively characterized in the central nervous system context [19,21,71,72], regarding essentially glutamate export, which also requires cyst(e)ine import. Herein, we observed that xCT expression in ES2 ovarian cancer cells is transcriptionally induced by cysteine and carboplatin (Figure 1C), with matching protein levels in cells exposed to both cysteine and carboplatin (Figure 1D). Interestingly, the same was observed for Nrf2 (Figure 1E,F), a major player in the cellular oxidative stress response and the main regulator of *xCT/SLC7A11* expression [23,29]. We, therefore, hypothesize that oxidative stress caused by drug exposure triggers Nrf2 expression and activity, while the resulting GSH depletion is indirectly responsible for increasing the Nrf2-regulated expression of genes involved in the antioxidant response. It was previously reported that Nrf2 dissociation from Kelch-like ECH-associated protein 1 (Keap1) and subsequent binding to antioxidant response element (ARE) regions (present in *xCT/SLC7A11* promoter region) are responsible for triggering a cytoprotective adaptive response [23,73,74,75]. Whether Nrf2 directly regulates xCT expression in ES2 and OVCAR3 cells needs further confirmation.

Notably, H_2_S metabolism is also mediated by Nrf2 via a regulatory loop. Whereas Nrf2 controls the expression of CBS, cystathionine γ-lyase (another major H_2_S synthase), and sulfide/quinone oxidoreductase (H_2_S-detoxifying enzyme), H_2_S activates Nrf2 via Keap1 inactivation through persulfidation of Cys_226_ and Cys_613_ [76]. Moreover, endogenous overexpression of CBS and the resulting increased H_2_S production in ovarian cancer cell lines are associated with metabolic reprogramming, mitochondrial morphogenesis, and chemoresistance [12,26,27,28,77]. In line with this Nrf2–Cys–H_2_S regulatory axis, increased xCT and Nrf2 expressions are fully compatible with our previous reports unraveling that ovarian clear cell carcinoma cells (ES2) are especially dependent on cysteine metabolism compared to ovarian serous carcinomas (OVCAR3), to cope with stressful conditions such as hypoxia and drugs [25,57].

As selenium is also transported by xCT [23,43], we hypothesized that selenium compounds are able to impair cysteine uptake. Albeit having antioxidant properties, selenium can also function as a pro-oxidant and exhibit toxicity toward tumor cells [40,42]. In the present study, we focused on the selenium–chrysin (SeChry) compound [42,48], which was described as having anti-tumor effects, even without selenium [42,48,49]. Despite ES2 and OVCAR3 cells exhibiting comparable sensitivity to SeChry-induced toxicity as determined by their EC_50_ values, the percentage of cell death upon exposure to 19 µM SeChry was higher in OVCAR3 than in ES2 cells. This is in agreement with our previous data showing that ES2 cells, when compared to OVCAR3, produce higher GSH levels and in a more efficient way upon exposure to drugs [22]. Notwithstanding, SeChry also induced cell death in non-malignant cell lines (Figure 2A–D), which could be explained by the recognized pro-oxidant role of selenium compounds, based on ROS generation, protein oxidation, and DNA binding, leading to impaired protein function, DNA damage, and cell death [42,48]. It was already shown that selenium compounds present antioxidant or pro-oxidant properties depending on their concentrations [45,78]. Interestingly, carboplatin abrogated SeChry cytotoxicity in ovarian cancer cells exposed to both compounds (Figure 2A,B). These results highlight that SeChry could be useful in the ovarian cancer clinical context but not if used concurrently with carboplatin administration. Strikingly, the protective effect of carboplatin upon SeChry exposure was not observed in non-malignant cells (Figure 2C,D), further indicating that SeChry is probably acting through different mechanisms in cancer and non-cancer cells. Another possibility is that carboplatin induces the cellular antioxidant responses in the malignant cells but not in the non-malignant ones, leading to a higher antioxidant response to SeChry. In addition to leading to cell death, SeChry increased xCT protein levels (Figure 2E), consistently with what was observed in other in vitro cancer models, associating a higher xCT activity with higher selenium salt (selenite) sensitivity [47]. These results are in line with what was previously reported about the association of SeChry cytotoxicity with changes in mitochondrial membrane potential [48], also consistent with increased oxidative stress. Indeed, whereas SeChry did not competitively inhibit the xCT-mediated cyst(e)ine uptake, it induced GSH degradation (Figure 4A–F) [15]. Accordingly, the extracellular concentrations of GSH and Cys-Gly, the first GSH degradation product, were generally increased in cells exposed to SeChry compared to control conditions (Figure 4C–F). Those increased extracellular GSH and Cys-Gly levels could, in turn, partially explain the decreased cell death observed upon the combined exposure of SeChry and carboplatin, as their extracellular chemical interactions could abrogate the effect of carboplatin and/or SeChry. Moreover, the Cys-Gly/GSH ratio (Figure 4G,H) shows that ES2 and OVCAR3 are equally subjected to GSH depletion. Collectively, our data strongly support the pro-oxidative role of SeChry in ovarian cancer cells (Figure 4) leading to GSH depletion. The reduction of GSH content observed mainly in OVCAR3 cells is in accordance with Martins et al. [48].

While the increased intracellular Cys availability observed in ES2 cells could sustain an increased production of the antioxidant H_2_S that could further counteract the pro-oxidant role of SeChry, the enzyme inhibition assays showed otherwise. Although we did not address SeChry effects in CBS inhibition in our cell models, we can speculate that this effect is stronger in ovarian cancer cells, as CBS was reported to be involved in the promotion of ovarian tumor growth, cisplatin resistance, and cellular bioenergetics [27], as well as in the regulation of mitochondria morphogenesis, promoting tumor progression in ovarian cancer [26]. Neatly, we unraveled that SeChry is a specific and relatively potent inhibitor of CBS. In fact, our data support that, while inhibiting CBS activity, 200 μM SeChry did not inhibit CSE or MST (Figure 5). CBS inhibition was suggested as an appealing strategy to combat chemoresistance in ovarian cancer [12,27,28], and as a generally valid anti-cancer approach [12,79]. In addition to the mechanistic relevance and pharmacological potential of SeChry in terms of additional therapeutic options for ovarian cancer, the discovery of a specific and relatively potent CBS inhibitor that does not inhibit CSE or MST should constitute a valuable research tool for the H_2_S field.

Despite the encouraging results obtained for SeChry against ovarian cancer cells, the observed cell death in non-malignant cells, HaCaT and HK2, is not desirable for an anti-cancer drug. To overcome this, we attempted a nanoparticle-based strategy. Nanoparticles are increasingly being used as drug carriers to increase the efficiency of delivery to cancer cells, thus minimizing their accumulation in surrounding tissues and the consequent side effects [80,81], while also decreasing chemoresistance [55,82]. Hence, we tested an encapsulated SeChry formulation, using polyurea dendrimers surface-functionalized with folate (SeChry@PURE_G4_-FA). Polyurea dendrimers are three-dimensional (3D) polymers bearing urea moieties in the backbone and peripheral amine groups, belonging to a family of water-soluble, biocompatible, biodegradable, pH-sensitive, intrinsically fluorescent polymers, presenting low toxicity in all studied generations (up to the sixth generation) without affecting cell viability [53]. As cancer cells express higher folate receptor levels [82,83], SeChry@PURE_G4_-FA may specifically deliver SeChry to ovarian cancer cells (ES2, OVCAR3, and OVCAR8), reducing toxicity in non-malignant cells. In fact, SeChry@PURE_G4_-FA induced significantly higher levels of cell death in ovarian cancer cells as compared to non-malignant cells (Figure 6). Notably, with this formulation, a reversion of SeChry toxicity upon carboplatin exposure was not observed in ovarian cancer cells, thus supporting its use along with platinum drugs when encapsulated. These different interactions of free SeChry versus encapsulated SeChry with carboplatin merits further attention in order to understand the mechanisms behind SeChry cytotoxicity abrogation by carboplatin.

## 5. Conclusions

**Figure 7:**
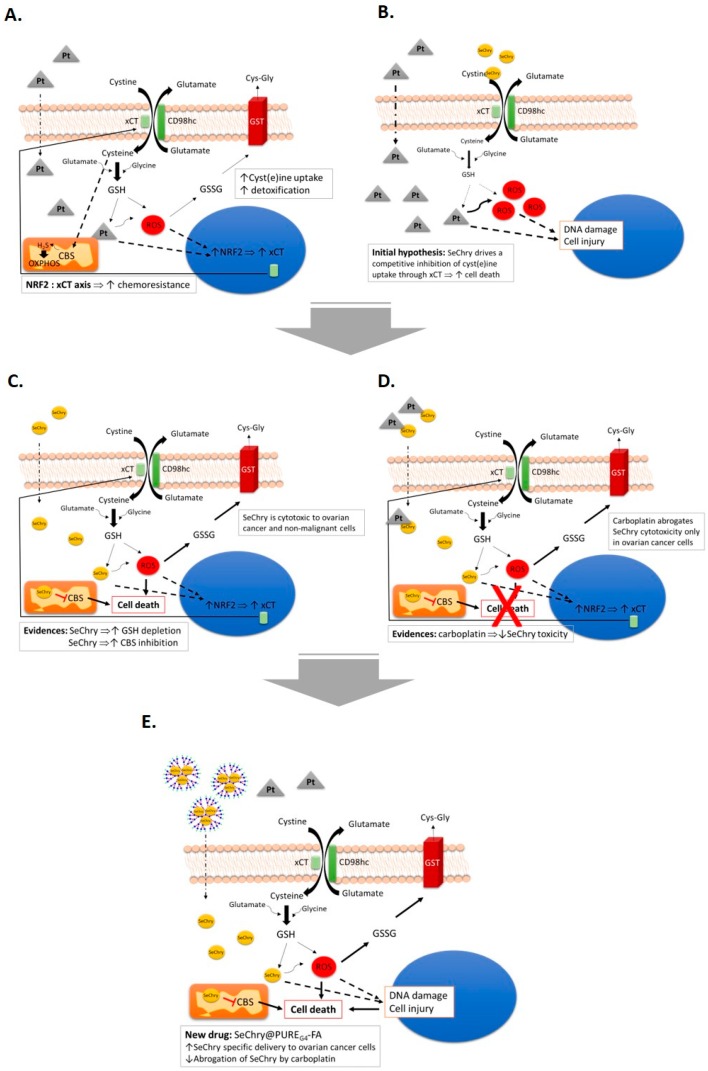
The central role of xCT transporter in chemoresistance and SeChry@PURE_G4_-FA as a new drug to treat ovarian cancer. (**A**) Putative effects of cyst(e)ine (cystine and cysteine) in platinum salt chemoresistance (carboplatin) by contributing to the bioavailability of glutathione (GSH), detoxifying drugs, and reactive oxygen species (ROS), contributing also to the production of H_2_S, supplying oxidative phosphorylation (OXPHOS). Oxidized GSH (GSSG) is exported through the action of glutathione transferase (GST) Exposure to carboplatin, putatively, activates *xCT*/*SLC7A11* transcription under the action of Nrf2. (**B**) We hypothesized that, by disturbing cyst(e)ine metabolic flux, chemoresistance would be reverted. Since the xCT transporter is also responsible for selenium uptake, we chose to test selenium-containing chrysin (SeChry) as a competitive inhibitor of xCT. (**C**) SeChry revealed to be toxic to ovarian cancer and non-malignant cells, albeit without impairing cyst(e)ine uptake. On the contrary, it increased GSH turnover and also inhibited cystathionine β-synthase (CBS), an H_2_S producing enzyme. (**D**) The cytotoxic effect of SeChry was abrogated by carboplatin in ovarian cancer cells, but not in non-malignant cells, probably due to the chemical interaction between the two compounds. (**E**) The selective delivery of SeChry encapsulated in a folate-targeted polyurea dendrimer nanoparticle (SeChry@PURE_G4_-FA) presents higher cytotoxicity to ovarian cancer cells compared to non-malignant cells and avoids SeChry inactivation by carboplatin. We propose SeChry@PURE_G4_-FA as a new drug formulation to treat ovarian cancer.

Collectively, our study supports SeChry@PURE_G4_-FA as a new targeted drug formulation with promising results in ovarian cancer treatment, a highly lethal disease largely due to the lack of an effective therapy. We disentangled two mechanisms of action of SeChry: increased oxidative stress prompting GSH depletion, and inhibition of the H_2_S-generating enzyme CBS, while upregulating xCT expression (Figure 7). Despite SeChry degradation not being observed in culture media, and other studies not describing deselenization for other selenium derivatives [84], the generation of SeChry diselenide derivatives and metabolites must be further investigated in order to fully characterize the metabolism of SeChry and the mechanisms underlying its cytotoxicity. 

## Supplementary Materials

The following are available online at https://www.mdpi.com/2072-6643/11/10/2523/s1, Figure S1: Determination of SeChry *EC_50_* for ES2 and OVCAR3 cell Lines. The effective concentration 50 (*EC_50_*) was determined based on the cell death levels induced by different concentration of selenium-chrysin (SeChry) in ovarian cell lines. Cell death was determined by flow cytometry, measuring annexin V-FITC and propidium iodide (PI) levels.
