# Supplementary material for: Targeting Glutathione and Cystathionine β-Synthase in Ovarian Cancer Treatment by Selenium–Chrysin Polyurea Dendrimer Nanoformulation
Source: Nutrients. 2019 Oct 19;11(10):2523. doi: 10.3390/nu11102523 (PMC6836284; doi:10.3390/nu11102523)
Supplement: Supplementary file 1 [file nutrients-11-02523-s001.pdf]

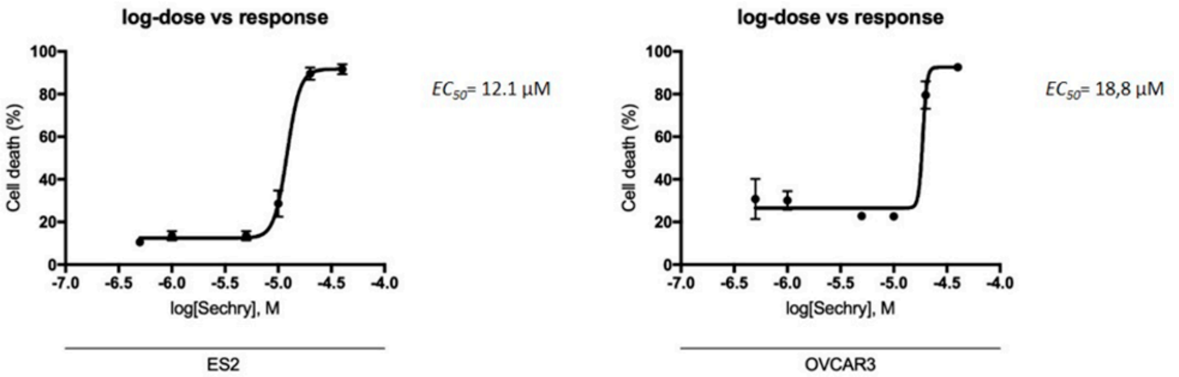

Figure S1. Determination of SeChry EC_{50} for ES2 and OVCAR3 cell Lines. The effective concentration 50 (EC_{50}) was determined based on the cell death levels induced by different concentration of selenium-chrysin (SeChry) in ovarian cell lines. Cell death was determined by flow cytometry, measuring annexin V-FITC and propidium iodide (PI) levels.
